# A numerical framework coupling finite element and meshless methods in sequential and parallel simulations

**DOI:** 10.1016/j.finel.2023.103927

**Published:** 2023-07

**Authors:** Van Dung Nguyen, Charlotte Kirchhelle, Amir Abdollahi, Julián Andrés García Grajales, Dongli Li, Kamel Benatia, Khariton Gorbunov, Sylvin Bielle, Alain Goriely, Antoine Jérusalem

**Affiliations:** a Department of Plant Sciences, University of Oxford, Oxford, UK; b Department of Engineering Science, University of Oxford, Oxford, UK; c Mathematical Institute, University of Oxford, Oxford, UK

**Keywords:** Finite element method, Meshless method, Parallel simulation, FE-MM coupling

## Abstract

The Finite Element Method (FEM) suffers from important drawbacks in problems involving excessive deformation of elements despite being universally applied to a wide range of engineering applications. While dynamic remeshing is often offered as the ideal solution, its computational cost, numerical noise and mathematical limitations in complex geometries are impeding its widespread use. Meshless methods (MM), however, by not relying on mesh connectivity, circumvent some of these limitations, while remaining computationally more expensive than the classic FEM. These problems in MM can be improved by coupling with FEM in a FEM-MM scheme, in which MM is used within sensitive regions that undergo large deformations while retaining the more efficient FEM for other less distorted regions. Here, we present a numerical framework combining the benefits of FEM and MM to study large deformation scenarios without heavily compromising on computational efficiency. In particular, the latter is maintained through two mechanisms: (1) coupling of FEM and MM discretisation schemes within one problem, which limits MM discretisation to domains that cannot be accurately modelled in FEM, and (2) a simplified MM parallelisation approach which allows for highly efficient speed-up. The proposed approach treats the problem as a quadrature point driven problem, thus making the treatment of the constitutive models, and thus the matrix and vector assembly fully method-agnostic. The MM scheme considers the maximum entropy (max-ent) approximation, in which its weak Kronecker delta property is leveraged in parallel calculations by convexifying the subdomains, and by refining meshes at the boundary in such a way that the higher density of nodes is mainly concentrated within the bulk of the domain. The latter ensures obtaining the Kronecker delta property at the boundary of the MM domain. The results, demonstrated by means of a few applications, show an excellent scalability and a good balance between accuracy and computational cost.

## 1 Introduction

The field of computational mechanics, fuelled by the widespread use of the Finite Element Method (FEM) and derivatives, has allowed for the treatment of complex problems in mechanics of materials and structures such as contact, impact/friction or blast [1–3], while also steadily gaining traction in biological sciences [4]. However, these particular problems often pose a significant challenge to computational modelling approaches: they are often non-linear and/or heterogeneous in terms of material composition, and can typically undergo large deformations. In such contexts, FEM with a Lagrangian description (often favoured for its computational efficiency) is non-ideal. Since the reference configuration is discretised by a finite element mesh, which is maintained during simulations, large deformations may result in excessive distortion of elements, in turn leading to simulation failure or unphysical results. These numerical problems can be overcome in different ways: by dissociating material and mesh motions, by making use of a remeshing technique, or by abandoning the mesh scheme.

In the first solution, FEM with a Eulerian description can be used. In a Eulerian formulation, the mesh is spatially fixed, thus naturally avoiding mesh distortion [5–7]. However, this approach suffers from difficulties in handling moving boundaries and in tracing history dependent behaviour. In order to avoid the disadvantages of both the Lagrangian and Eulerian formulations, FEM with an Arbitrary Lagrangian–Eulerian description (known as “ALE”) has been proposed [8,9] to combine the best features of pure Lagrangian and Eulerian analyses.

The second possibility to avoid mesh distortion is to consider remeshing strategies. When a Lagrangian finite element mesh becomes too distorted, a new mesh is created to improve local accuracy where required [10–12]. However, the results need to be transferred from the old mesh to the new mesh. Hence refinement schemes are often computationally expensive.

In the last option, abandoning the mesh scheme leads to a so-called Meshless Method (MM). MMs construct the approximation of unknowns from a cloud or neighbourhood of nodes without mesh connectivity, thus relaxing the strong tie between mesh topology and approximation quality in FEM [13]. These methods thus appear ideal for modelling problems in which the domain suffers from large deformations, moving boundaries or material growth. A wide variety of MMs employing different approximation and solution schemes has already been proposed in the literature, including the Smoothed Particle Hydrodynamics (SPH) [14], the least-square collocation meshless method [15], the Optimal Transport Method (OTM) [16], the Natural Element Method (NEM) [17,18], the Reproducing Kernel Particle Method (RKPM) [19], the MeshLess local Petrov–Galerkin method (MLPG) [20], the Partition of Unity Finite Element Method (PUFEM) [21], the Hp-clouds [22], the Element-Free Galerkin (EFG) [23,24], among many others. A comprehensive review about the MMs can be found in Ref. [13].

Among a wide variety of MMs employing different approximation and solution schemes, the so-called Galerkin MMs based on the weak form of the problem’s partial differential equation, and in particular, the Element-Free Galerkin method (EFG) using moving least square (MLS) approximations [23,24], have been extensively and successfully adopted [23,25–27]. The FEM and EFG schemes share the same Galerkin weak form, allowing either FEM, MM, or a coupled FEM-MM to be performed within the same code. However, while the FEM shape functions are constructed using the Lagrange interpolation within each particular element and satisfy the Kronecker delta property, the MM shape functions are usually built by fitting methods with a group of nodes located in the influence domain and generally do not satisfy the Kronecker delta property. A comprehensive review about the interpolation scheme can be found in Ref. [28]. Here, we employ EFG with maximum entropy (max-ent) approximation scheme [29,30], in which the weak Kronecker delta property is obtained on convex parts on the boundary of a MM domain, i.e., shape functions of internal nodes vanish on convex parts of the boundary. In particular, we focus on the direct coupling not only between the FEM and EFG domains but also inbetween EFG domains by leveraging the weak Kronecker delta property of the max-ent shape functions. The FEM-EFG coupling avoids mesh distortions by employing MM domains in high deformation regions and FEM domains in the remaining (less distorted) regions, while the EFG-EFG coupling facilitates the parallel implementation of the MM scheme, in which the interpolation scheme can be found independently in each processor.

Despite its superiority in terms of accuracy and convergence [23,31], EFG and other MMs are still computationally more expensive than FEM due to complex interpolations and numerical integrations. Two strategies have been used to ameliorate the computational costs associated with MM schemes.

Firstly, costs can be reduced by coupling FEM and MM discretisation schemes to use MM within sensitive regions (i.e., those that undergo large deformations), while retaining more efficient FEM for other less distorted regions. The major difficulty is to satisfy the continuity of the displacement field on the interface between the domains of two methods. There exist different approaches for a coupled FEM-MM scheme. Interface elements have been considered between the FEM region and the EFG region using MLS shape functions, and hybrid shape functions combining both MLS and FEM shape functions are used for these transition elements [32]. The continuous blending method was proposed in Ref. [33,34] using a mixed hierarchical approximation. Lagrange multipliers were used in Ref. [35–37] to constrain the continuity of the displacement at interfaces between FEM and MM regions. The collocation approach was introduced in Ref. [38] to couple FEM and EFG regions by constraining the continuity of the unknown field at each node at FEM-MM interfaces. A review of the FEM and MM coupling can be found in Ref. [39]. Direct coupling without transition or interface elements between a FEM region and a MM region using the max-ent shape functions was also proposed in Ref. [40–42] by exploiting their weak Kronecker delta property. However, the direct coupling between the displacement field satisfying the Kronecker delta property in the FEM region with the displacement field only satisfying the weak Kronecker delta property in the MM region a priori leads to a discrepancy of the displacement field at the FEM-MM interface. Note that FEM-MM coupling can also be performed weakly by using a range of different techniques, e.g., Lagrange multipliers [43,44], mortar contact [45,46], and Nitche’s method [47,48].

Secondly, computational efficiency in FEM simulations of large domains is often increased by implementing parallelisation strategies that allow simulations to be run on multiple processors rather than sequentially. Such strategies have also been implemented for EFG methods [49–51], however parallelisation is a non-trivial problem due to the treatment of adjacent nodes from neighbouring regions that fall within the domain of influence or neighbourhood of a given quadrature point or node. In previous frameworks, such nodes have been duplicated to maintain computational accuracy at intra-domain boundaries [49] by preserving domains of influence equivalent to those in comparable sequential simulations. While effective, this strategy is also suffering from additional computational costs in comparison to parallelisation schemes for FEM, that only require to share nodes directly at the domain boundaries. Ideally, one would want a “ghosting” parallel coupling strategy somehow leveraging the weak Kronecker property to link MM subdomains (between each other or with FEM domains) similarly to what is achieved between FEM subdomains.

The main problem in coupling FEM and MM (within a coupled FEM-MM scheme) and coupling MM and MM (within a MM scheme in parallel) occurs at the boundary between these domains, where the Kronecker delta property is not satisfied for the MM domains. In this work, we propose a direct FEM-MM and MM-MM coupling avoiding any discrepancy in the real displacement of the shared boundary nodes at the FEM-MM and MM-MM interfaces, respectively. A key ingredient to guarantee the accuracy in this coupling strategy is the locality of the shape functions of the MM domains at the FEM-MM or MM-MM interfaces. For this purpose, the MM domains consider max-ent shape functions, and two different strategies are proposed by leveraging the weak Kronecker delta property of the max-ent shape functions in the MM domains:

- The MM domain needs to be convex at the interfaces with other domains to obtain the weak Kronecker delta property at those parts of its boundary. Indeed, in a convex MM domain using maxent shape functions, the internal nodal shape functions do not contribute to a point that lies on the boundary of this convex domain, and the approximation at each boundary node of this domain thus only receive contributions from neighbouring boundary nodes [30]. It will be shown that although this simplification leads to a discrepancy in the real displacement of the shared boundary nodes at the interfaces with other domains, simulations are still accurate and stable.
- To isolate the contributions of the boundary nodes, we propose a simple and effective strategy based on a structured mesh refinement on the interior nodes, i.e., each boundary node of the MM domain corresponds to a vertex node of a convex polytope. It is noted that at a vertex node of a convex polytope, the Kronecker delta property is satisfied [30]. Consequently, a true Kronecker delta property at the boundary of a MM domain is obtained, leading to a seamless transition at the interfaces with the MM domains. When this structured mesh refinement is employed, the convexity of the MM domains is no longer required. It is noted that this strategy leads to a larger system to be solved since the mesh is refined in the MM domains.

The proposed coupled scheme does not limit the class of domains that can be studied. Regarding the decomposition into FEM and MM domains in a coupled FEM-MM scheme, identifying MM regions undergoing large deformation for a given problem in a couple FEM-MM scheme is not a trivial task. This procedure often relies on a preprocessing stage, and these regions are fixed during simulation. Since such a region is generally not known a priori, deciding on appropriate FEM and MM regions in a given problem relies either on the user experience or on a simulation with coarse mesh in which the regions with high deformations can be identified. To overcome these problems, an automatic adaptive FE-MM coupled method was proposed [42,52]. In this work, we manually choose MM domains. An automatic FEM-MM domain decomposition will be considered in future work.

Here, we present a numerical framework that combines the benefits of FEM and MM to study large deformation problems without heavily compromising on computational efficiency. The programme is modular offering a variety of different solvers and constitutive models [53], which can be further adapted and developed depending on user needs. Therefore, the proposed solution is an ideal environment to tackle large deformation problems with bespoke computational approaches. In this work, we primarily focus on the different discretisation schemes available, namely FEM, MM (EFG method), and coupled FEM-MM. Similar to other recent work [49], we employ a maximum entropy (max-ent) approximation scheme for the EFG regions within the domain. In addition to assessing the computational efficiency and enhanced accuracy of coupled FEM-MM simulations in our framework, we also implement parallelisation for large domain simulations. In contrast to previous approaches [49], we employ a simplified strategy that does not consider the influence of nodes outside the partition assigned to each processor, but convexifies the subdomains to leverage the scheme’s weak Kronecker delta property. We demonstrate that despite this simplification, simulations are still accurate and stable, and parallelisation leads to highly efficient speed-up within our MM framework. Furthermore, we provide evidence that small computational inaccuracies at the boundary between two processors can be eliminated simply through a structured remeshing strategy concentrating the higher nodal density to the bulk of the subdomain.

## 2 General framework

We consider a general boundary value problem under large strains. A body *Ω*_0_ ⊂ ℝ^*d*^, where *d* is the dimension of the problem, undergoes a deformation characterised by the mapping ***φ*** (***X***) **: *X*** ∈ *Ω*_0_ → ***x*** ∈ *Ω*, where *Ω* denotes the deformed configuration. The displacement field is defined as

(1) u(X)=x−X=φ(X)−X.

The body *Ω*_0_ is subjected to a force per unit mass ***b*** and to boundary conditions at its boundary *∂Ω*_0_, which includes two non-overlapping parts: a Dirichlet part *∂*_*D*_*Ω*_0_ where the displacement field ***u*** is prescribed by $\bar{u}$, and a Neumann part *∂*_*N*_ *Ω*_0_ where the traction is prescribed by $\bar{t}$. The equilibrium equations stated in the material form are given as follows:

(2) $$\nabla_{0}\cdot P+\rho b-\rho \ddot{u}=0\;\text{in}\;\Omega_{0}\;,$$

(3) $$u-\bar{u}=0\;\text{on}\;\partial_{D}\Omega_{0}\;,\;\text{and}\;$$

(4) $$P\cdot N-\bar{t}=0\;\text{on}\;\partial_{N}\Omega_{0}\;,$$

where **∇**_0_ is the gradient operator with respect to the reference configuration, ***P*** is the first Piola–Kirchhoff stress tensor, *ρ* is the material density, and ***N*** is the outward unit normal on *∂*_*N*_ *Ω*_0_. In order to solve for the displacement field ***u***, the constitutive relationship must be introduced to complete the problem statement^2^ :

(5) P(t)=𝒫(F(τ)withτ∈[0,t]),

where ***F*** = **I** + **∇**_0_ ⊗ ***u*** is the deformation gradient with **I** being the second-order identity tensor and **𝒫** is the material operator. It is noted that the modelling framework proposed in the remaining of the paper does not require any assumptions on the constitutive relationships.

To solve the boundary value problem stated by Eqs. (2)–(5), the proposed approach provides maximum flexibility in spatial discretisation, allowing either FEM, MM, or a coupled FEM-MM within the same problem. To maximise efficiency, parts of domain where local deformation is not significant are modelled with FEM, while the regions with important distortion are making use of MM. Independently of the numerical approximants used (i.e., FEM or MM), the conservation of linear momentum (2) is rewritten under the following weak form:

(6) $$\int_{\Omega_{0}}P:\nabla_{0}⊗\delta u\;\text{d}V+\int_{\Omega_{0}}\rho \overset{..}{u}\cdot \delta u\;\text{d}V=\int_{\Omega_{0}}\rho b\cdot \delta u\;\text{d}V+\int_{\partial_{N}\Omega_{0}}\bar{t}\cdot \delta u\text{d}S,$$

for all admissible virtual displacements *δ****u***. Either by FEM or by MM, the domain *Ω*_0_ is discretised into a set of *N* discrete nodes. At an arbitrary material point ***X*** ∈ *Ω*_0_, ***u*** and *δ****u*** are approximated through the usual discretisation as:

(7) $$u(X)=\sum_{a=1}^{n}\Phi_{a}(X)u_{a}\;\text{and}\;\delta u(X)=\sum_{a=1}^{n}\Phi_{a}(X)\delta u_{a},$$

which are typically evaluated at quadrature points during integration and assembly. In Eq. (7), *n* is the number of nodes participating to the approximation, *a* refers to the nodes in the FEM or MM neighbourhood of ***X***, *Φ*_*a*_ is the corresponding shape function, and ***u***_*a*_ (and *δ****u***_*a*_) is the vector of unknowns (and virtual counterpart) at node *a*. In the case of FEM, the “neighbourhood” of a node is the list of nodes sharing an element with it, while the “neighbourhood” of a quadrature point (or Gauss point in the case of FEM) is the list of nodes forming the element containing said quadrature point (as explained later, a background mesh is used to identify those points in MM domains). In the proposed approach, the weak form (6) is solved in a unified manner as a quadrature point driven problem, thus making the FEM, MM, and couple FEM-MM schemes only different at the preprocessing stage, where the “neighbourhoods” of each quadrature point are formed. Using Eq. (7), the weak form (6) leads to a system of non-linear equations of the nodal unknowns, which is solved using the conventional Newton–Raphson iterative process [54].

For first-order consistency, the shape functions *Φ*_1_, …, *Φ*_*n*_ need to satisfy at every point ***X*** ∈ *Ω*_0_:

(8) $$X=\sum_{a=1}^{n}\Phi_{a}(X)X_{a}\;\text{and}\;\sum_{a=1}^{n}\Phi_{a}(X)=\;1,$$

in which any affine function is exactly reproduced by the approximation scheme. A comprehensive review about the interpolation scheme can be found in Ref. [28]. One important aspect of an interpolation scheme expressed by Eq. (7) is the Kronecker delta property, i.e., *Φ*_*a*_(***X***_*b*_) = *δ*_*ab*_. In the FEM context, the Kronecker delta property is always satisfied, leading to ***u (X_a_)*** = ***u_a_***, while in the MM context, the Kronecker delta property is not satisfied in general, leading to ***u*** (***X***_*a*_) ≠ ***u***_*a*_. As a result, the imposition of the Dirichlet boundary conditions in MM is not as straightforward as in FEM and requires further treatments [23,24,55,56]. When the MM scheme employs max-ent shape functions, the weak Kronecker delta property is observed, i.e., the approximation at each boundary node of the boundary of a convex MM domain receives only contributions from neighbouring boundary nodes [30]. As a result, the issue of applying Dirichlet boundary conditions vanishes when the prescribed value is uniform.

### 2.1 FEM

The shape functions of FEM interpolate the solution between discrete values obtained at mesh nodes, which are determined by the mesh types and function orders. In general, the coordinates ***X*** of the mesh element are mapped into isoparametric coordinate system (*ξ, η, ζ*) for simplified calculation of shape functions *Φ* (see, e.g., Ref. [57] for details):

(9) $$X=\sum_{a=1}^{n}\Phi_{a}(\xi ,\eta ,\zeta )X_{a}\;\text{and}\;\sum_{a=1}^{n}\Phi_{a}(\xi ,\eta ,\zeta )=1,$$

where *n* is the number of nodes of the element containing ***X*** and the definition of shape function *Φ*_*a*_ is determined by the order of polynomials used for the functions. Note that the classical Lagrangian FEM shape functions only satisfies *C*^0^-continuity, though higher order continuity shape functions have been proposed [58]. In the MM scheme, the higher continuity can be naturally obtained by defining the suitable shape functions.

### 2.2 MM

The difference between FEM and MM arises from the way the approximation of the unknown field is made using the weak form (6). In this work, instead of the MLS approximation scheme [24], we employ max-ent shape functions [29,30,59]. In contrast to MLS, the max-ent approximation can produce a weak Kronecker delta property on the boundary of a convex MM domain, i.e., the shape functions of the interior nodes do not contribute to the approximation of the displacement field at those boundary nodes, while the Kronecker delta property is satisfied at the vertices of the domain. A recent study [60] has shown that the max-ent was more accurate by a factor of two compared to the MLS for irregular node distributions in problems undergoing high deformations. The weak form (6) is integrated in MM domains by using the background mesh obtained by triangulation of the cloud of nodes, in turn facilitating the choice of quadrature points as Gauss points of this resulting mesh. In the numerical examples, 6 integration points for the triangles and 4 integration point for tetrahedra of the MM background mesh are used. Note that other quadrature techniques can reduce the computational cost of numerical integration in MMs, see, e.g., Ref. [61].

The theory of the max-ent shape functions is briefly introduced. Each shape function *Φ*_*a*_(***X***) is defined as the probability of influence of a node *a* at space ***X***. The max-ent formulation is: find the set of the shape functions ***Φ*** as the solution of the constrained optimisation problem:

(10) $$\begin{array}{l} \max\left[H(\Phi ,m)=-\sum_{a=1}^{n}\Phi_{a}(X)\ln\left(\frac{\Phi_{a}(X)}{m_{a}(X)}\right)\right],\\ \Phi_{a}(X)\geq 0,\\ \sum_{a=1}^{n}\Phi_{a}(X)=1,\\ \sum_{a=1}^{n}\Phi_{a}(X)X_{a}=X, \end{array}$$

where the shape functions *Φ*_*a*_(***X***) depend on the prior function *m*_*a*_(***X***) used. The main advantage of this approach is that the internal nodal shape functions do not contribute at a point that lies on the boundary of the domain, thus simplifying the imposition of the essential boundary conditions; in particular, at the vertices of a convex domain, the Kronecker delta property is thus naturally verified, and essential boundary conditions can be applied directly on the node. The prior *m*_*a*_(***X***) can be uniform (the contributions of influences are treated equally among nodes in the neighbourhood) or non-uniform (nodes are assigned with different influential weights such as Gaussian weight, cubic or quadratic weights), which gives greater flexibility in the construction of new approximants [59]. Here, we choose a cubic spline weight function *m*_*a*_(***X***) ≡ *m (q*_*a*_) with *m* defined as

(11) $$m(q)=\{\begin{array}{ll} \frac{2}{3}-4q^{2}+4q^{3} & \;\text{if}\;\;0\;⩽\;q\;⩽\;\frac{1}{2},\\ \begin{array}{l} \frac{4}{3}-4q+4q^{2}-\frac{4q^{3}}{3}\\ 0 \end{array} & \begin{array}{l} \text{if}\;\frac{1}{2}\;⩽\;q\;⩽\;1,\\ \text{otherwise,} \end{array} \end{array}$$

where $q_{a}=||X-X_{a}||/r_{a}^{\max}$ ‖ ⋅ ‖, is the *L*^2^-norm, and $r_{a}^{\max}$ is nodal weight function support radius. This support domain size is defined as $r_{a}^{\max}=\alpha h_{a}$, where *α* is a dimensionless parameter, and *h*_*a*_ is the “nodal spacing” chosen here as the *L*^2^-norm average of the three Euclidean distances to the three closest nodes to node *a* (see Ref. [59]). Therefore, the shape functions become sharper and more local as the nodal spacing *h*_*a*_ decreases.

The set of the nodal weight function support radius $R=\left[r_{1}^{\max},\ldots ,r_{N_{\text{node}\;}}^{\max}\right]=\alpha \left[h_{1},\ldots ,h_{N_{\text{node}\;}}\right],$, where *N*_node_ is the number of nodes, characterises the degree of locality of the basis functions and has a strong effect on the accuracy of the numerical solutions. As the value of *α* increases, the shape functions become less local. The effect of *α* on the numerical solutions is considered in the next section.

Defining ***λ*** as the vector of Lagrange multipliers *λ*_*s*_ (*s* = 0, 1, 2, …, *d*) associated with *d* + 1 constraints, where *d* is the dimension of the problem, the shape functions can be written as follows:

(12) $$\begin{array}{l} \Phi_{a}(X)=\frac{Z_{a}(X)}{Z(X)},\\ Z(X)=\sum_{a=1}^{n}Z_{a}(X),\\ Z_{a}(X)=m_{a}(X)\exp\left(-X_{a}^{T}\lambda (X)\right), \end{array}$$

where *Z*_*a*_ (***X***) depends on the type of compact support used.

Finally, the dual problem consists in finding ***λ*** such that:

(13) λ=argminln(Z(X,λ)).

Such unconstrained optimisation problems can be solved with different numerical algorithms such as steepest descent, Newton’s method, quasi-Newton methods and interior-point methods. The F90 libraries of MAXENT-F90 (version 1.4) were interfaced with the programme to provide the corresponding shape functions (http://dilbert.engr.ucdavis.edu/~suku/maxent/). Choosing a “good” value of *α* is not a trivial task. A too small value of *α* leads to a limited number of nodes inside the support radius and makes the optimisation problem ill-posed and unable to converge. A too large value results in a large linear system to be solved as the band of the stiffness matrix depends on the support radius. In this work we propose a simple adaptive strategy of *α* as follows:

(i) Start with an initial value *α*_0_ of *α*, e.g., *α*_0_ = 1;
(ii) Perform the optimisation procedure to compute the shape functions (and their gradients) at each integration point. If the optimisation solution at one quadrature point cannot be achieved, go to (iii); otherwise, go to (iv);
(iii) Assign *α* = *γα* where *γ* > 1 is constant; go to (ii);
(iv) End.

After several iterations, a suitable value of *α* can be obtained. In this work, when this adaptive strategy is applied, the values *α*_0_ = 1 and *γ* = 1.1 are used.

### 2.3 Parallel implementation

The partition of the model mesh (or nodes) is implemented through two mechanisms: domains can either be manually assigned to each processor (in either FEM, MM, or coupled FEM-MM), or partitioning is performed automatically through external libraries such as METIS, an open source software package for partitioning large meshes [62]. The former case allows for the possibility to enforce the topological convexity of each MM partition (METIS being graph-based does not necessarily verify convexity), leading to a weak Kronecker delta property of the MM shape functions. This means that the internal nodal shape functions do not contribute to a point that lies on the boundary of the partition and that boundary nodes thus only receive contributions from neighbouring boundary nodes, while vertex nodes, however, verify the Kronecker delta property [59]. As discussed later, this key property will be leveraged to couple FEM and MM schemes assigned to different partitions. When a mesh partitioning is automatically performed by METIS, only the FEM mesh is partitioned, and all the MM nodes are assigned to one processor by default (as METIS does not guarantee convexity). This approach can lead to a deterioration of parallel scaling if the MM processor tasks dominate the FEM tasks. One alternative is to manually assign to each processor convex MM domains as shown in Section 3.4.

**Fig. 1:**
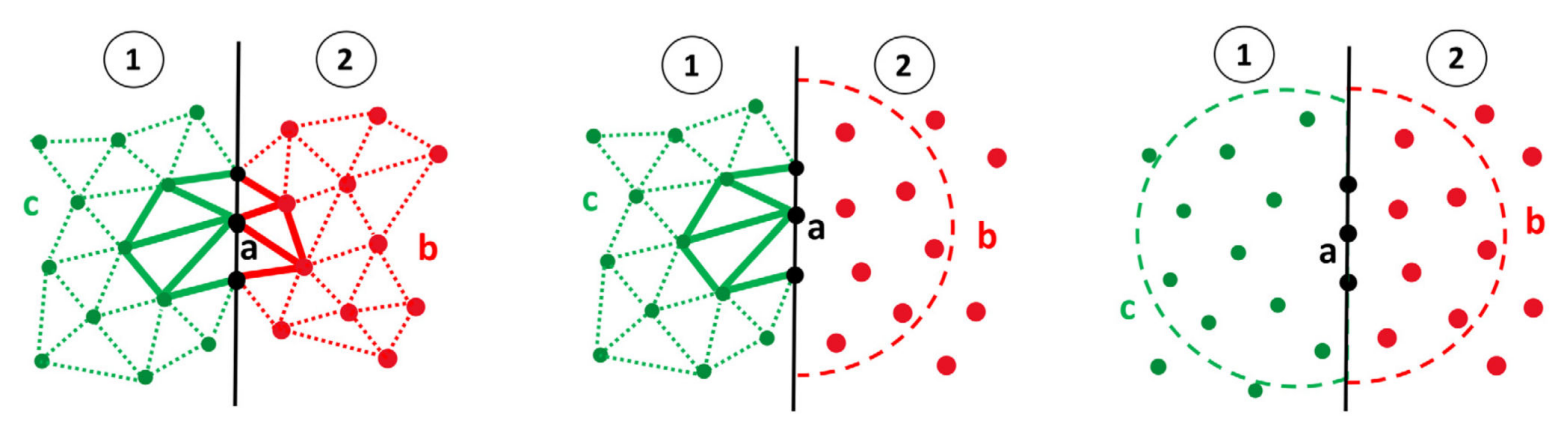
Schematic of two adjacent partitions where (left) both partitions are assigned to FEM with arbitrary element distributions of nodes *b* and *c*, (middle) the left partition is assigned to FEM with an arbitrary element based distribution of nodes *c* and the right partition is assigned to MM with an arbitrary distribution of nodes *b*, (right) both partitions are assigned to MM with arbitrary distributions of nodes *b* and *c*. In all cases, the black nodes *a* represent the shared nodes over the boundary (line) between the two partitions. The elements with bold continuous lines indicate the elements which contain the middle shared node. The semicircle dashed line represents the neighbourhood of the middle shared node in MM.

The main problem in coupling FEM and MM (or two MM domains) occurs at the boundary between partitions where only weak delta property is observed for the MM subdomains. Indeed, as one does not necessarily have *Φ*_*a*_(*ξ*_*a*_, *η*_*a*_, *ζ*_*a*_) = 1 for boundary node *a*, the MM interpolation scheme can lead to the following inequality: ***u***(***X***_*a*_) ≠ ***u***_*a*_, i.e., the stored nodal unknown ***u***_*a*_ of the discretisation scheme is not the “real” displacement of the node at ***X*** = ***X***_*a*_, but what we term here a “fictitious nodal displacement”. The true displacement needs to be interpolated along with the other nodes in the neighbourhood of the node of interest to obtain its real displacement. This is obviously not the case in FEM, where the stored value is also the real nodal displacement. Fig. 1 shows the schematic of two adjacent partitions with a common node *a* on the boundary for three cases: FEM and FEM, FEM and MM, and MM and MM. Here, we make the choice (i) to not allow the neighbourhood of a node in a MM partition to reach into an adjacent partition, and (ii) to carry on the parallelisation scheme by solving the discretised nodal unknown displacement the same way as it is done in FEM, i.e., taking $u_{a}^{f(1)}=u_{a}^{f(2)}$, where the superscript *f* (*i*) refers to the fictitious value in partition *i*. In the following, we explore the implications of this choice.

When considering both partitions, the real values of displacement $u_{a}^{r}$ at the shared node *a* is obtained as a function of their respective neighbourhood nodal values of ***u***^*f*^ in the first and second partitions following:

(14) $$u_{a}^{r(1)}=\sum_{b=1}^{n_{1}}\Phi_{b}^{\left(1\right)}\left(X_{b}\right)u_{b}^{f(1)}\;\text{and}\;u_{a}^{r(2)}=\sum_{b=1}^{n_{2}}\Phi_{b}^{\left(2\right)}\left(X_{b}\right)u_{b}^{f(2)},$$

respectively, where *n*_1_ and *n*_2_ are the numbers of nodes in the neighbourhood of node *a* for each partition. For the simulation to be sound, the continuity of real displacement is a priori required, i.e., $u_{a}^{r(1)}=u_{a}^{r(2)}$. Here, for computational efficiency, we make the choice to enforce the fictitious displacements instead, i.e., $u_{a}^{f(1)}=u_{a}^{f(2)}$. In the case of assigning FEM to both partitions (Fig. 1-left), as the FEM shape functions satisfy the Kronecker delta property, i.e., $\Phi_{a}^{\left(1\right)}\left(X_{a}\right)=\Phi_{a}^{\left(2\right)}\left(X_{a}\right)=1$, Eq. (14) naturally leads to $u_{a}^{r(1)}=u_{a}^{f(1)}=u_{a}^{f(2)}=u_{a}^{r(2)}$ (and the two neighbourhoods reduce to the set of nodes of the elements containing node *a* in each partition). However, this continuity and the equality between fictitious and real displacements do not hold when at least one of the partitions is assigned to MM (Fig. 1-middle and -right) since the MM shape functions do not satisfy the Kronecker delta property, except at the vertices of a convex domain [59]. Previously, this problem has been addressed by creating a broadened zone of shared nodes at the boundary [49], which in turn diminishes the scalability of the parallel scheme. The following section details a simple yet effective strategy to avoid this issue.

The computation under parallel implementation is supported by PETSc [63–65], a suite of data structures and routines for the scalable solution for partial differential equations. PETSc governs the communication, assembly, and disassembly of the local implementation from each processors.

### 2.4 Mesh refinement

**Fig. 2:**
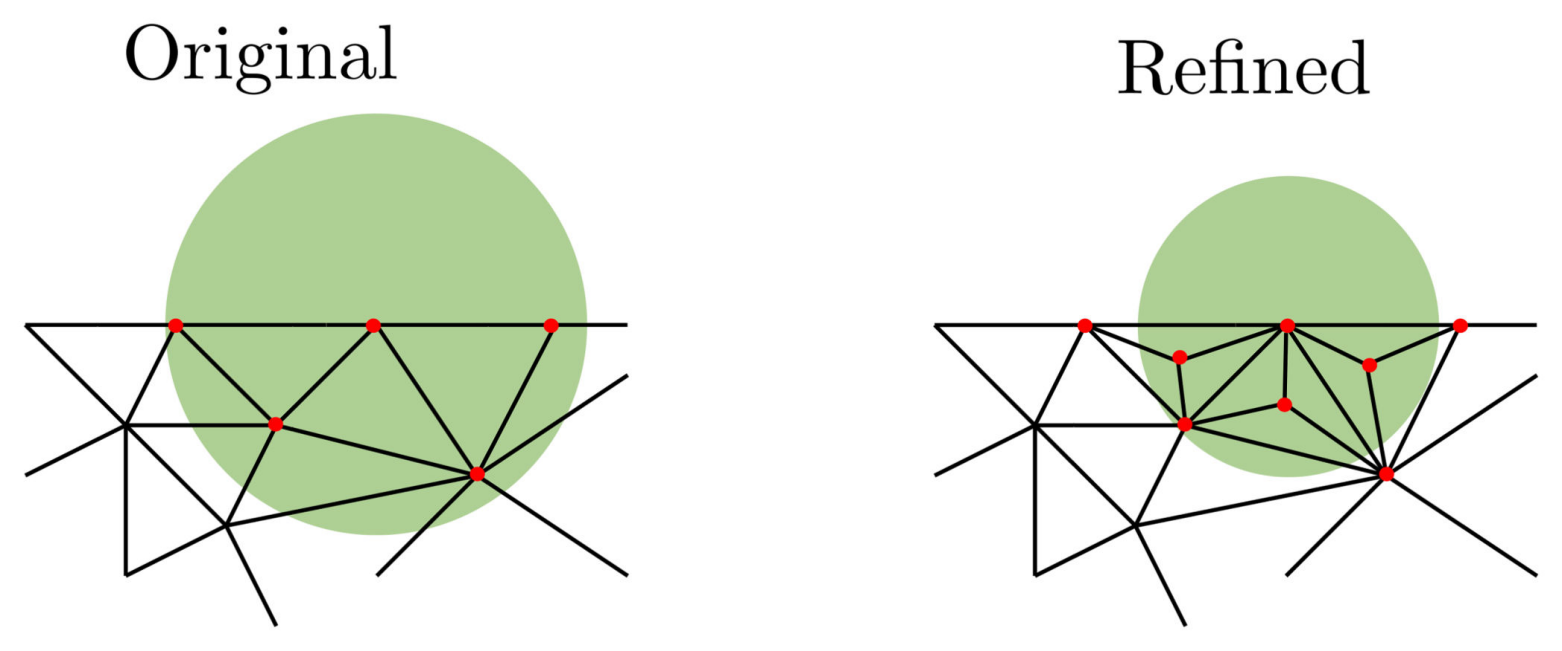
Mesh refinement strategy to minimise the discrepancy in the real displacement of the shared boundary nodes. A support domain at a node at the MM boundary (shaded in green) is represented. In the original mesh (left), the support domain includes multiple boundary nodes, whereas in the refined mesh (right), a smaller support radius can be obtained, leading to less boundary nodes inside the support domain.

Up to this point, the framework has ignored the discrepancy in real displacement of the shared boundary nodes at the FEM-MM or MM-MM interfaces (e.g., in parallel simulations). A key ingredient to guarantee the simulation accuracy is the locality (controlled by the support radius) of the shape functions of the nodes at the boundaries of MM parts. A large support size generally produces unreliable results due to the influence of neighbouring boundary nodes for each node of the interfaces (in particular in non-convex domains) while a small support size is constrained by the MM domain under consideration to form a well-defined problem. Pragmatically, one can expect the existence of an optimal support domain size. Here, we propose a simple and effective strategy based on a structured mesh refinement on the interior nodes to isolate the contributions of the boundary nodes, i.e., each boundary node of the MM domain corresponds to a vertex node of a convex polytope, at which the Kronecker delta property is satisfied [30,59], see Fig. 2. Consequently, a true Kronecker delta property at the boundary of a MM domain can be obtained, leading to a seamless transition at the FEM-MM and MM-MM interfaces.

This approach results in a higher interior nodal density around the boundary nodes under consideration. Support domains for the refined cloud of nodes using this strategy were slightly smaller than in the original mesh while including comparable numbers of nodes. Thanks to the reduction of the support domain size, support domains of boundary nodes avoid including neighbouring boundary nodes, shifting the relative contribution from the boundary to the bulk. The mesh refinement strategy can be performed at all elements inside each MM domain or only at the elements sharing nodes with the MM boundaries. However, the former increases significantly the number of nodes (and thus the size of the finite element system) while the latter allows for a better accuracy at very little additional computational cost, see Section 3.3.

## 3 Numerical examples and discussion

### 3.1 Mesh convergence in a coupled FEM-MM scheme

**Fig. 3:**
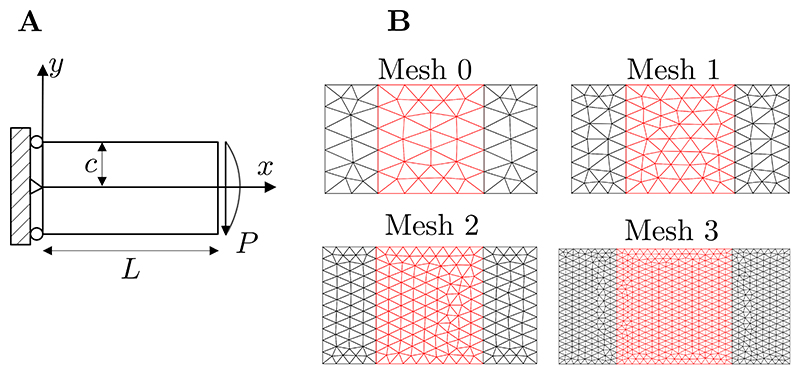
Linear elastic cantilever beam loaded by a parabolic distribution of tractions at the end. (A) Geometry and boundary conditions. (B) Different meshes used in the error analysis with mesh size. The red region in the middle of the specimen corresponds to the MM region in the cases the coupled FEM-MM is considered. When MM is used, these meshes are used as background meshes.

In order to evaluate the efficiency of the coupled FEM-MM simulations, an error analysis is carried out by considering the standard benchmark problem of a linear elastic cantilever beam loaded by a parabolic distribution of tractions at the end, see Fig. 3A. The exact solution for this problem is known (see, e.g., Ref. [66]) as follows

(15) $$\{\begin{array}{l} u_{x}=\frac{Py}{6EI}\left[(6L-3x)x+(2+v)\left(y^{2}-c^{2}\right)\right],\;\text{and}\;\\ u_{y}=-\frac{P}{6EI}\left[3vy^{2}(L-x)+(4+5v)c^{2}x+(3L-x)x^{2}\right] \end{array},$$

where *L* and *c* are respectively the length and the half width of the specimen, see Fig. 3A, $I=\frac{2c^{3}}{3}$, *P* is the total applied force, and *E* and *v* are, respectively, the Young’s modulus and the Poisson’s ratio. In order to characterise the error of the simulation in comparison with the exact solution, the relative *L*_2_ and *H*_1_ norms of the displacement field are considered to evalute the error; with

(16) $$\varepsilon_{L_{2}}=\frac{{\left|\text{u}-{\text{u}}^{\text{exact}\;}\right|}_{L_{2}}}{{\left|{\text{u}}^{\text{exact}\;}\right|}_{L_{2}}}\;\text{and}\;\varepsilon_{H_{1}}=\frac{{\left|\text{u}-{\text{u}}^{\text{exact}\;}\right|}_{H_{1}}}{{\left|{\text{u}}^{\text{exact}\;}\right|}_{H_{1}}},$$

where **u** and **u**^exact^ are, respectively, the simulated and exact solutions, and the *L*_2_ and *H*_1_ norms are defined by

(17) $$\begin{array}{l} |\text{a}{|}_{L_{2}}=\sqrt{\int_{{\text{Ω}}_{0}}\text{a}\cdot \text{a}dV}\;\text{and}\;\\ |\text{a}{|}_{H_{1}}=\sqrt{\int_{{\text{Ω}}_{0}}\left(\text{a}\cdot \text{a}+L^{2}\left(\text{a}⊗\nabla_{0}\right):\left(\text{a}⊗\nabla_{0}\right)\right)dV}, \end{array}$$

for a vector field **a** and where *Ω*_0_ is the domain under investigation. The problem is discretised with linear triangular elements. Four different meshes are considered, see Fig. 3B. When MM is employed, these meshes are used as the background meshes (to define the quadrature points and for visualisation purposes), while the mesh nodes are the MM nodes. Doing so allows us to keep, for a given mesh, the same number of degree of freedom independently of the resolution scheme under consideration (FEM, MM, and coupled FEM-MM). When FEM-MM is employed, MM is considered in the middle region, while FEM is considered in the remaining regions. The values *L* = 3 m, *c* = 1 m, *E* = 2 × 10^3^ Pa, *v* = 0.3 and *P* = 150 N are arbitrarily used in this analysis.

**Fig. 4:**
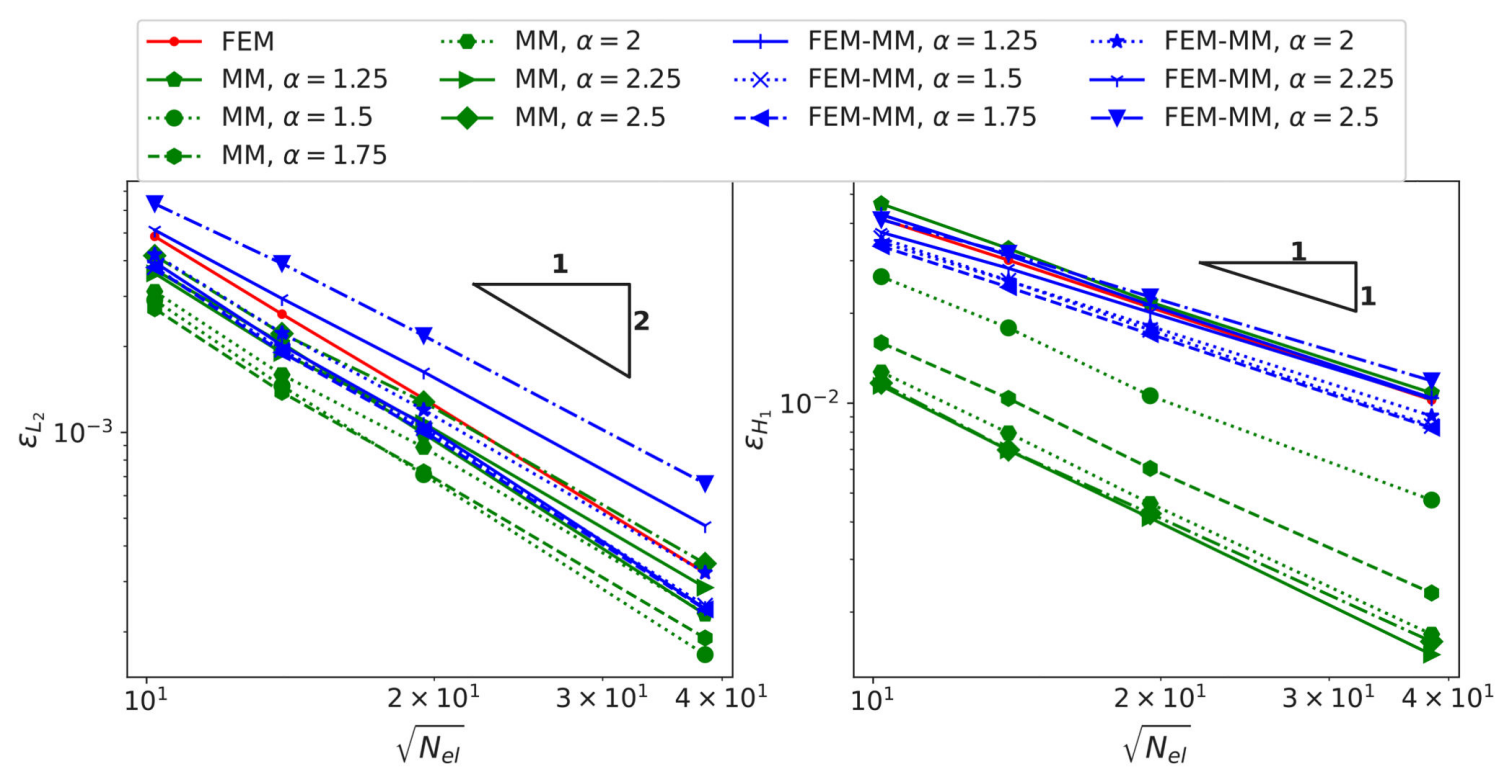
Convergence of the *L*_2_ error (left) and of *H*_1_ error (right) with respect to the number of elements (*N*_el_) for different values of the scaling factor *α*. Note that for low values *α* (e.g., close to 1), the solution for the preprocessing optimisation required to establish the shape functions cannot be achieved, and only the results with the values of *α* starting from 1.25 are reported.

The convergences of the relative *L*_2_ error $\left(\varepsilon_{L_{2}}\right)$ and of the relative *H*_1_ error $\left(\varepsilon_{H_{1}}\right)$ with respect to the number of elements *N*_*el*_ are shown in Fig. 4 with different values of the scaling factor *α*. When MM is employed, it can be seen that the accuracy of the numerical solution measured in the *H*_1_ norm can significantly change depending on the locality of the shape functions, as controlled by *α*. The less local the function, the more accurate the solution up to a limit, as generally observed in MM. For high values of *α*, the accuracy in *L*_2_ error is degraded, which could be corrected by using a more accurate, though more computationally expensive, quadrature [67]. Generally, MM provides more accurate results than FEM with a large enough value of *α*. When the coupled FEM-MM scheme is employed, the solution is less accurate than a sole MM scheme for the same discretisation, but is more accurate than FEM with small values of *α* (1.25, 1.5, 1.75 and 2). For larger values of *α* (2.25 and 2.5), as a larger number of neighbouring nodes are obtained as a result of a larger support radius, the difference between the true displacement field and the fictitious one as mentioned in Section 2.3 is more important in the FEM/MM interfaces, which degrades the accuracy of the FEM-MM scheme. However, this mismatch can be minimised using the mesh refinement strategy detailed in Section 3.3.

**Fig. 5:**
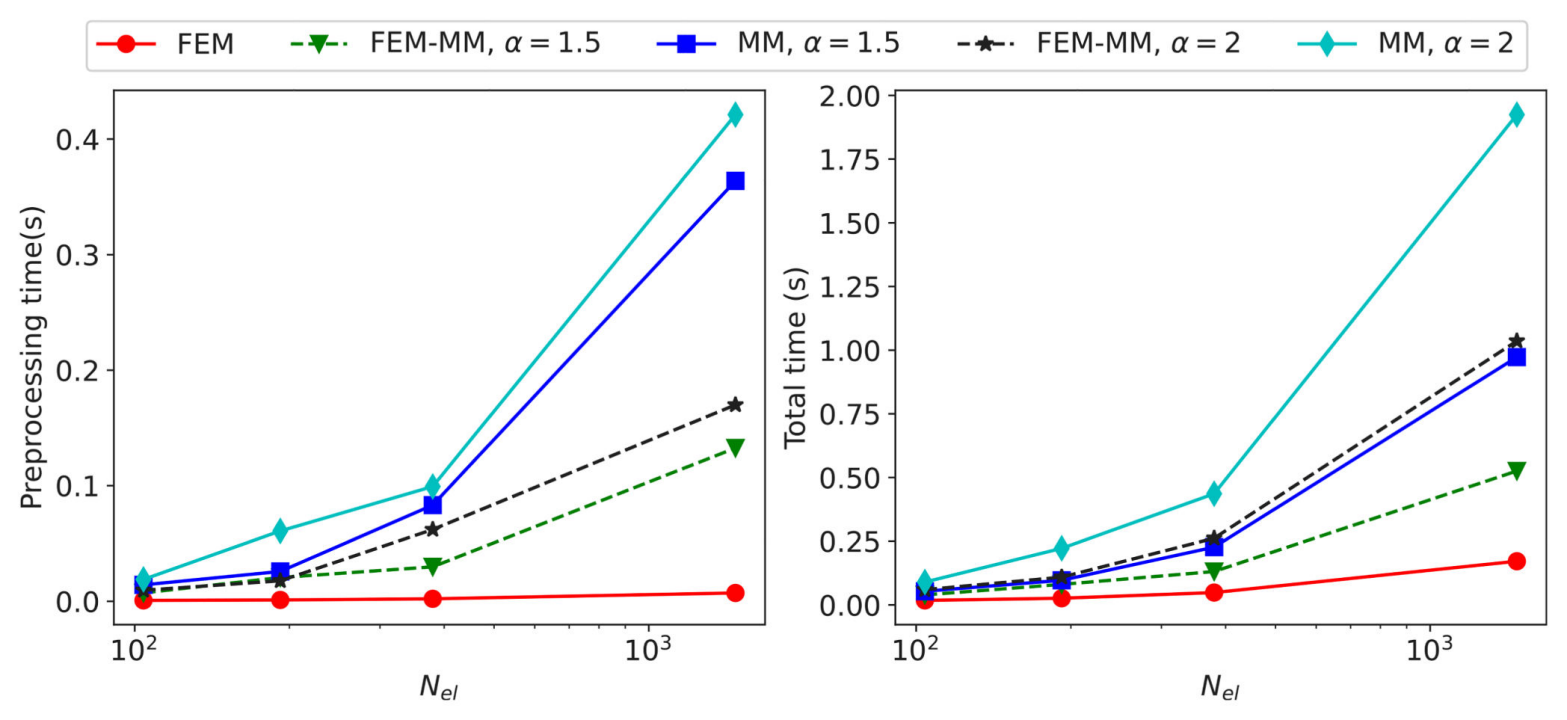
Computational time when using FEM, MM, and coupled FEM-MM with respect to the number of elements in the (background) meshes for the cases *α* = 1.5 and *α* = 2 reported in Fig. 4. The preprocessing time refers to the time needed for reading the input file and establishing the shape functions at each integration point.

The comparison of the computation time between FEM, MM, and the coupled FEM-MM schemes are reported in Fig. 5 with two typical values of *α*: 1.5 and 2. The preprocessing time refers to the time needed for reading the input file and establishing the shape functions at each integration point. The preprocessing time is very small compared to the total one in the case of FEM but considerable in the cases of MM and FEM-MM. However, the FEM-MM requires less preprocessing time than MM as the optimisation step needed to establish the shape functions can ignore the FEM domains. The total time for MM increases quickly with the number of elements and with *α*. A larger value of *α* results in a larger non-zero band in the stiffness matrix, making the solution more computationally expensive. However, the coupled FEM-MM scheme is more computationally efficient than the MM alone.

**Fig. 6:**
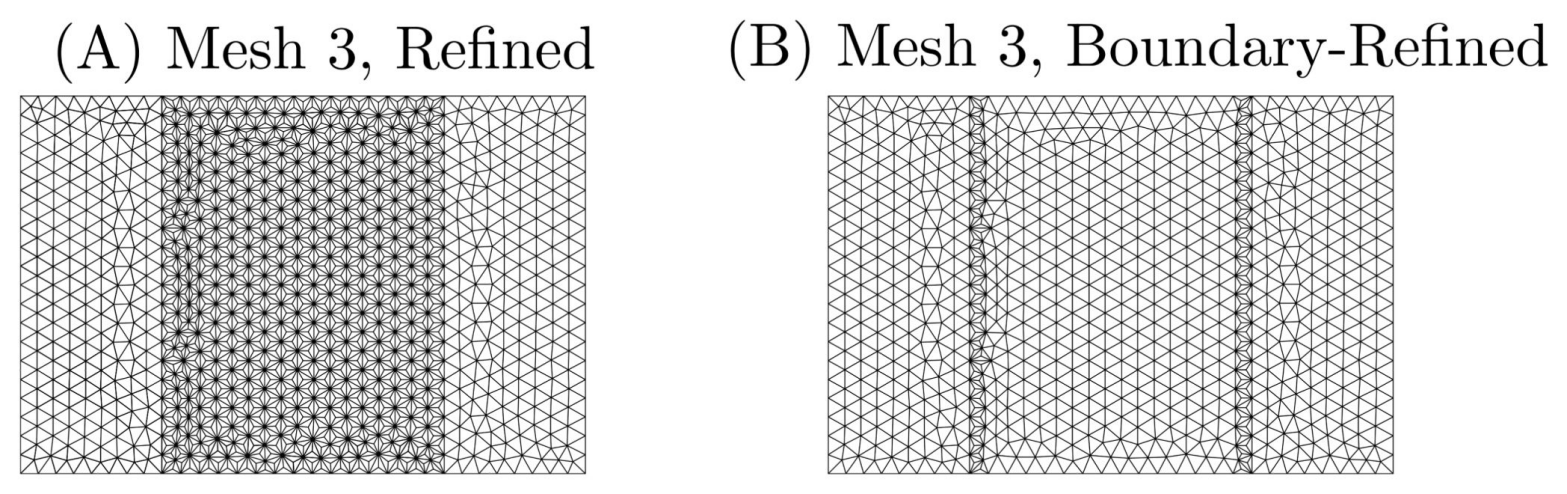
Mesh refinement performed in all elements of the MM domain (A) and at boundaries of the MM domain (B). Only the case “Mesh 3” reported in Fig. 3 is shown for illustration purposes.

**Fig. 7:**
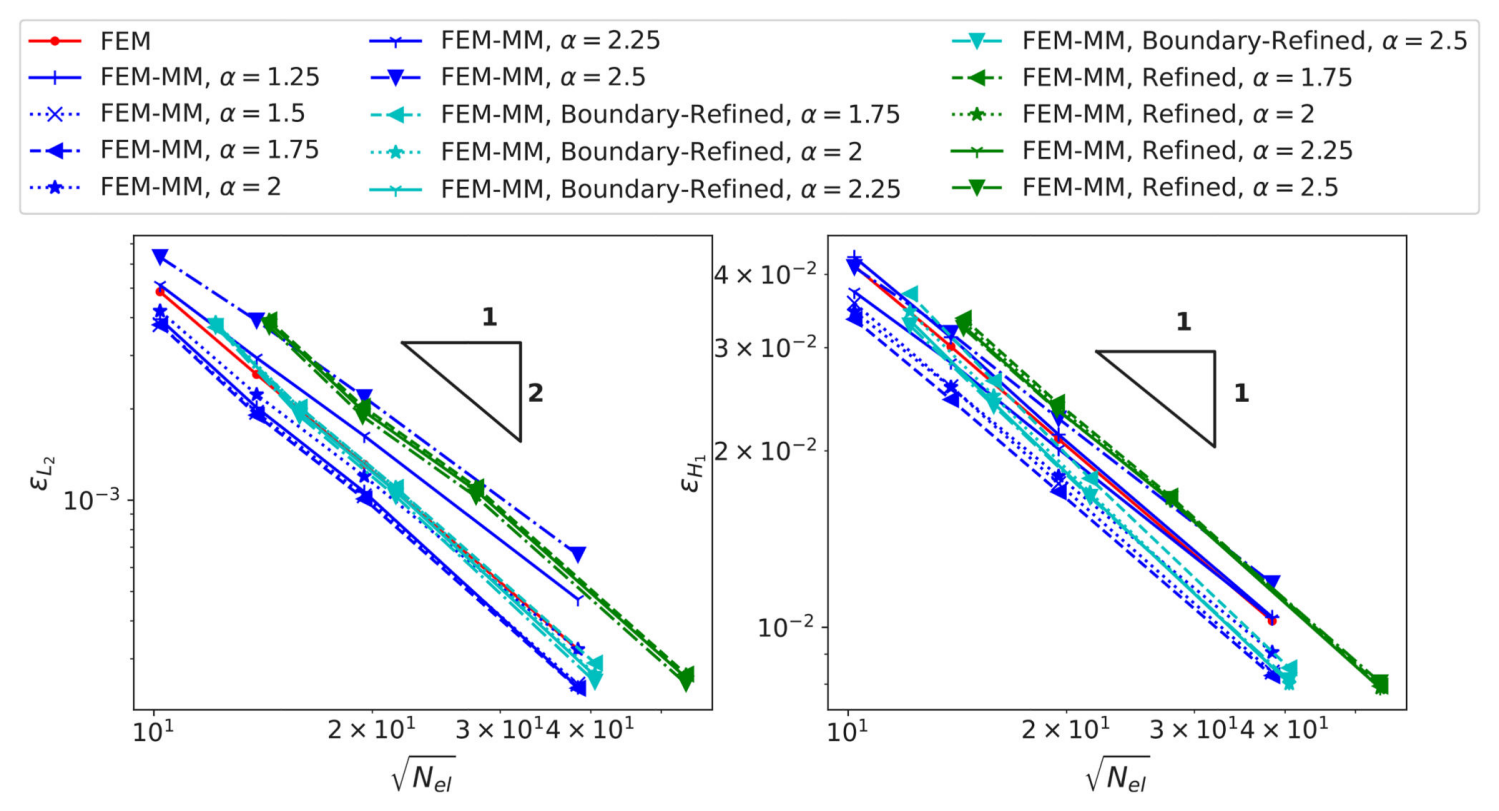
Convergence of the *L*_2_ error (left) and of *H*_1_ error (right) with respect to the number of elements (*N*_el_) for different values of the scaling factor *α* when using mesh refinement strategies. The results without mesh refinement are also reported for comparison purposes. Note that for low values *α*, the solutions for the preprocessing optimisation required to establish the shape function cannot be achieved; those results are not reported.

The efficiency of the mesh refinement strategy described in Section 2.4 is investigated. Two different scenarios are considered as shown in Fig. 6: (i) “Refined” when the remeshing is performed on the whole MM domain and (ii) “Boundary-Refined” when the remeshing is performed only on the element having shared nodes with the FEM-MM interfaces. The convergences of the relative *L*_2_ error $\left(\varepsilon_{L_{2}}\right)$ and of the relative *H*_1_ error $\left(\varepsilon_{H_{1}}\right)$ with respect to the number of elements *N*_*el*_ are shown in Fig. 7 with different values of the scaling factor *α*. Results for values of *α* in which the optimisation procedure to compute the shape function does not converge are not shown. It can be seen that mesh refinement allows for an improvement of the accuracy. Although the number of nodes significantly increases when the mesh refinement strategy is applied to the whole mesh, the accuracy of the numerical solution is almost similar to the case with the mesh refinement strategy performed at the elements sharing nodes at the FEM-MM interfaces.

### 3.2 Extension of a hyperelastic block

**Fig. 8:**
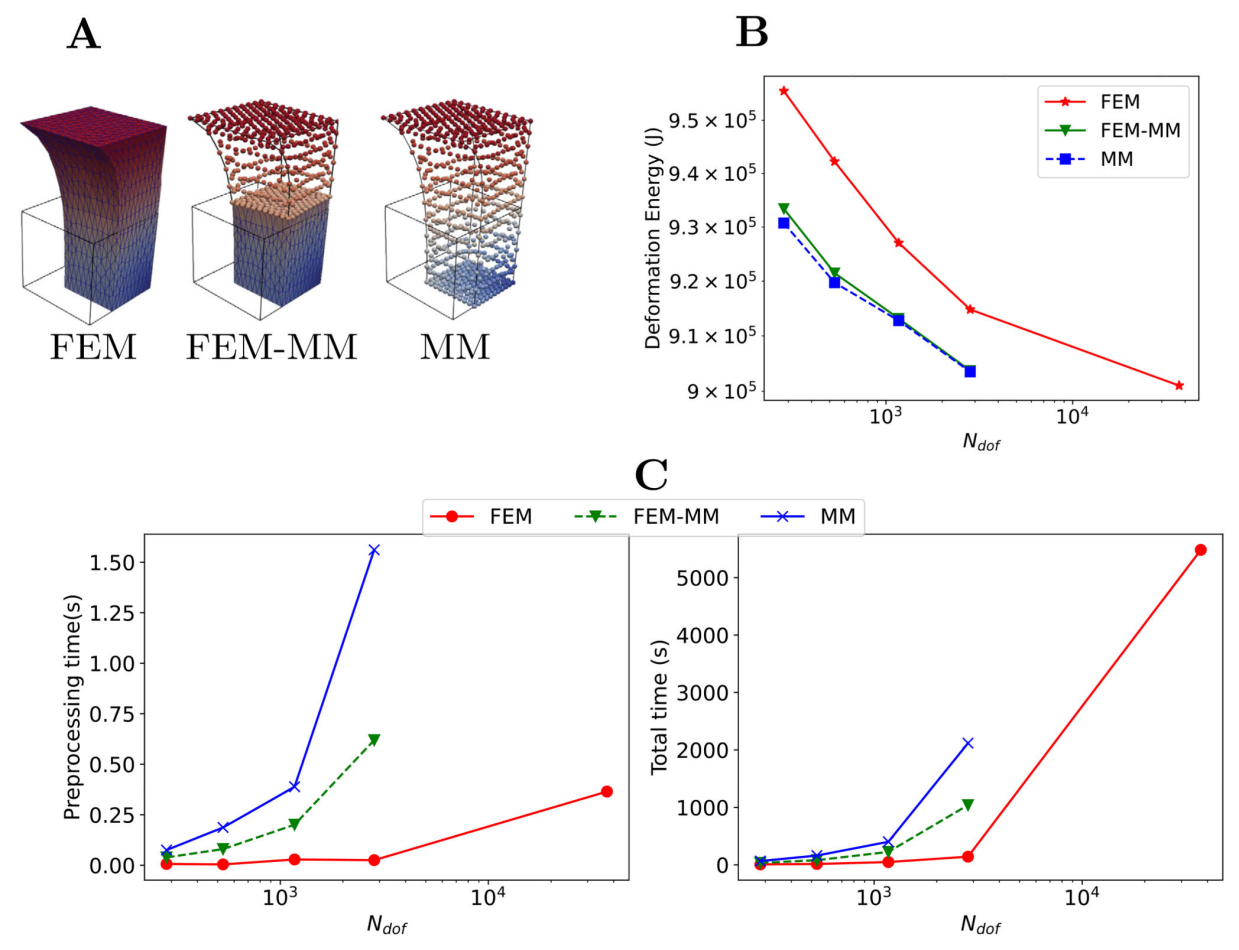
Extension of a hyperelastic cube. (A) Deformed shape at 100% extension in the case of FEM, MM and FEM-MM. (B) Dependence of the total deformation energy with respect to the number of degrees of freedom. (C) Preprocessing and computational time with respect to the number of degrees of freedom. The preprocessing time refers to the time consumed for reading the input file and establishing the shape functions at each integration point.

In this section, we demonstrate the performance of the coupled FEM-MM in a strongly nonlinear problem. A hyperelastic block is stretched vertically with the top and bottom faces prescribed in all directions. As a result of the symmetry, it is sufficient to model one eighth of the block, in which the top face is fixed, two adjacent side faces traction-free and symmetry boundary conditions applied on the rest of the faces, see Fig. 8A. A similar test was performed in Ref. [30]. The material is a compressive neo-Hookean with an energy density defined as

(18) $$\Psi (F)==\frac{1}{2}\lambda \ln^{2}J-\mu \ln J+\frac{\mu }{2}\text{tr}(F^{T}\cdot F),$$

where ***F*** is the deformation gradient tensor, *J* = det ***F*** is the Jacobian, and $\lambda =\frac{Ev}{\left(1+v)(1-2v\right)}$ and $\mu =\frac{E}{2(1+v)}$ are the Lamé constants with *E* and *v* being, respectively, the Young’s modulus and the Poisson’s ratio. In this test, *E* = 10^6^ Pa and *v* = 0.485 are used. A value close to the incompressibility limit is considered as the finite element solution in this case converges very slowly and coarse meshes result in very large errors [30] while MM shows a much better accuracy for the same node discretisation. The cube is deformed up to 100% extension in 100 loading increments.

For the purpose of showing the efficiency of the FEM-MM scheme, the adaptive strategy to find the value of *α* described in Section 2.2 is used here. Five different meshes are considered consisting of 444, 823, 1946, 5028 and 72 550 linear tetrahedral elements. The first four meshes are considered in the cases of FEM, MM and FEM-MM while the finest mesh is only considered with FEM. When the coupled FEM-MM scheme is employed, the cube is divided into two parts: the lower part as FEM and the upper part as MM, see Fig. 8A, since the mesh distortion is more important in the latter. Fig. 8A shows the deformed configurations of the block at 100% tensile deformation when using the finest mesh. Fig. 8B shows the dependence of the total elastic energy with respect to the number of degrees of freedom in the cases of FEM, MM and FEM-MM schemes. Fig. 8C shows the increasing computational time for each simulation upon mesh refinement. It can be seen that the total deformation energy decreases monotonically with mesh refinement. The total deformation energies in the MM and coupled FEM-MM simulations remain close and are always smaller than the one of FEM for a given nodal discretisation, implying improved accuracy as observed in Ref. [30]. To achieve a total elastic energy comparable to the best MM case, a very fine element mesh needs to be considered for a much higher computational time, see Fig. 8C. The computational time of the FEM-MM scheme is found in-between the ones of MM and of FEM schemes. Clearly, the use of the coupled FEM-MM scheme allows for an improvement of the solution accuracy at a lower computational cost compared to the MM scheme

### 3.3 Parallelisation of the MM scheme

While combining FEM and MM schemes can accomplish a significant saving in simulation run time compared to a MM scheme alone, combined schemes may not be practical in cases where significant parts of the structure in question undergo large deformations. An alternative strategy to optimise simulation time is the parallelisation of the simulation, in which the model is partitioned into distinct regions which are run in parallel on multiple processors.

**Fig. 9:**
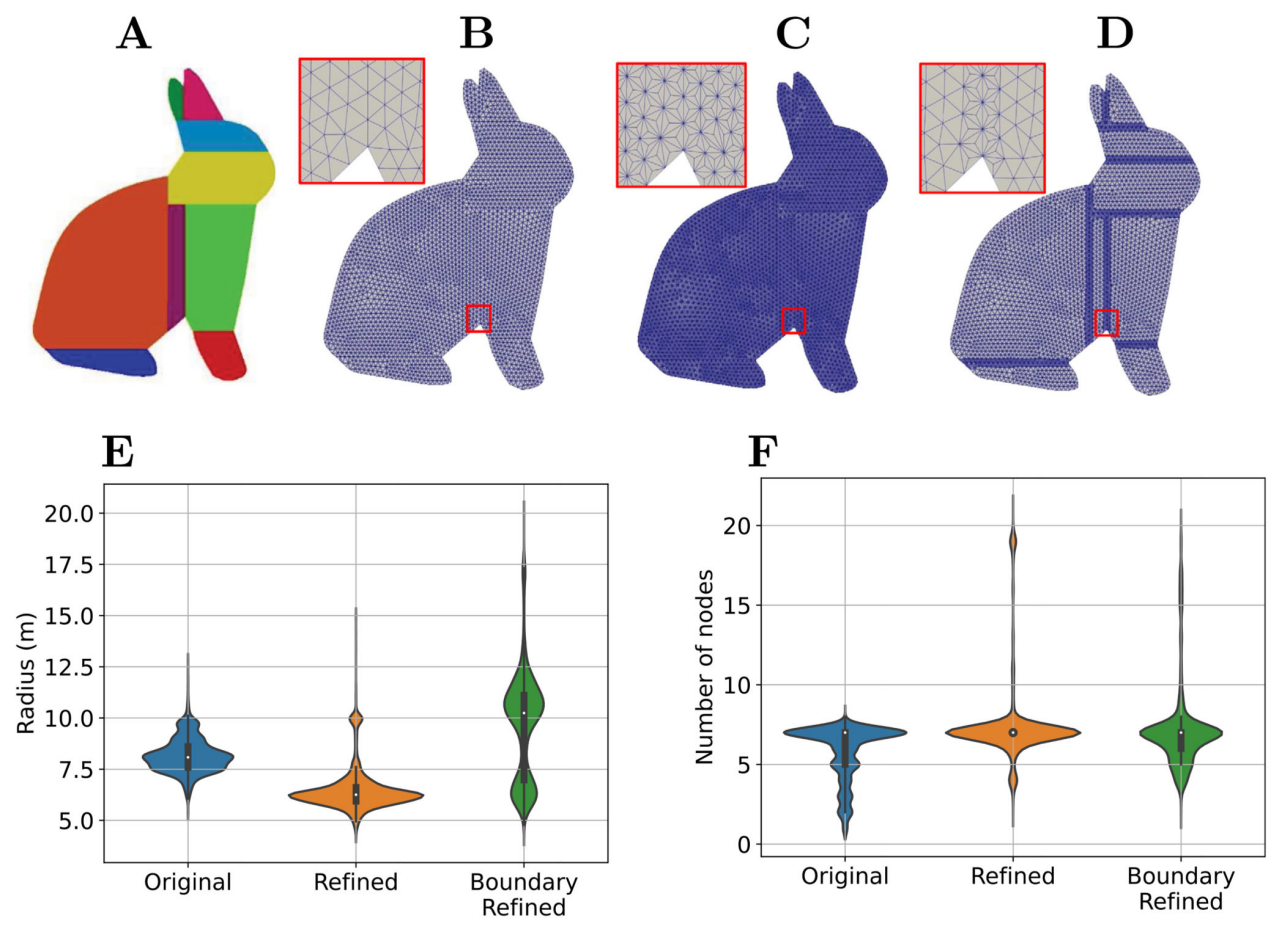
Parallelisation of the MM scheme. (A) 2D Stanford bunny partitioned into 9 convex regions using V-HACD. (B, C, D) Nodal distribution and mesh geometry in Original (B), Refined (C), and (D) Boundary-Refined cases. (E, F) Violin plots of support domain radius (E) and number of nodes within each support domain (F) for the Original, Refined, and Boundary-Refined Standford bunny shown in B, C and D, respectively.

In this section, a 2D version of the Stanford bunny was “convexified” and partitioned using Volumetric Hierarchical Approximate Convex Decomposition (V-HACD) [68]. This scheme allows to create a number of convex subdomains from any geometry, by modifying it slightly to facilitate the process, as a full convex partitioning of the Bunny would lead to a much larger and unworkable number of subdomains, see Ref. [68] for more details. Flaws in the mesh were repaired using ICEM-CFD from the Ansys suite (https://www.ansys.com). The final mesh was decomposed into 9 convex domains (Fig. 9A), and contained 2607 nodes and 4979 linear triangular elements (Fig. 9B). The Refined and Boundary-Refined strategies are shown in Fig. 9C and D, respectively. The adaptive strategy to find the value of *α* described in Section 2.2 is used. Support domain sizes were modified in the refined and boundary-refined cases as compared to the ones of the original mesh (Fig. 9E) while including comparable numbers of neighbouring nodes (Fig. 9F).

**Fig. 10:**
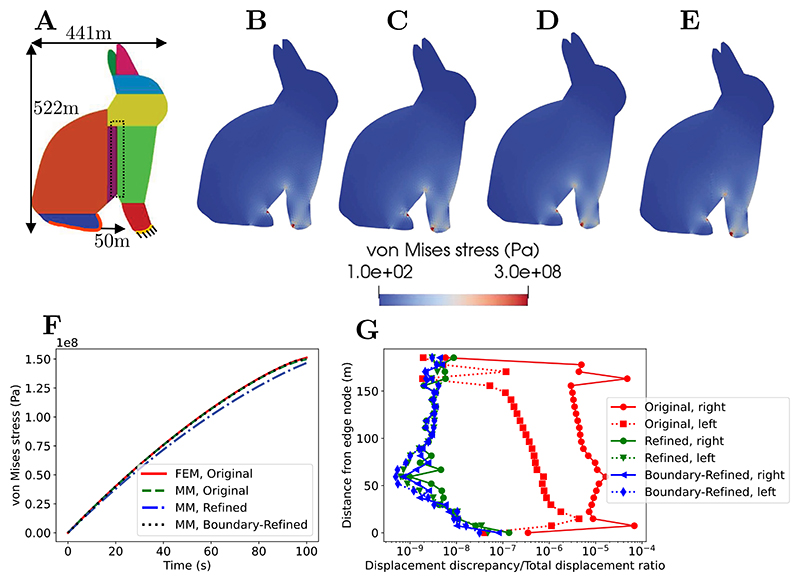
Effect of remeshing strategy in the parallel MM scheme. (A) Stanford bunny dimensions and loading. (B) FEM simulation of original mesh, (C, D, E) MM simulations of Original, Refined, and Boundary-Refined cases. (F) Average von Mises stress of the five elements sharing the vertex node at the boundary between the bunny front leg and belly in the Original mesh, or the equivalent area in the refined simulation, see Fig. 9. (G) Ratio between the magnitude of the fictitious-real displacement discrepancy to real displacement magnitude at shared nodes between processors in the compressed region behind the foreleg (see dash box in A). “Left” and “right” of lines refer to the two subdomains on both sides of the boundary.

In the simulation, the bunny’s height was (arbitrarily) taken to be 522 m, the front leg of the Bunny was fixed in the *x*- and *y*-directions, and the hindleg pulled forward 50 m over an arbitrary time of 100 s (the problem being rate-independent, the time is of no specific relevance) to create a region with highly compressive forces between both legs (Fig. 10A). The material was taken as a quasi-incompressible hyperelastic St-Venant-Kirchhoff model following:

(19) $$\begin{array}{c} P=F\cdot \mathbb{C}:\frac{1}{2}\left(F^{T}\cdot F-I\right),\\ \;\text{with }{\mathbb{C}}_{ijkl}=\frac{Ev}{\left(1+v)(1-2v\right)}\delta_{ij}\delta_{kl}+\frac{E}{2(1+v)}\left(\delta_{ik}\delta_{jl}+\delta_{il}\delta_{jk}\right),\; \end{array}$$

where ℂ is the fourth order Hooke tensor, and *E* and *v* are respectively the Young’s modulus and Poisson’s ratio; ***P*** is the first Piola–Kirchhoff stress tensor. The values *E* = 10^9^ Pa, *v* = 0.3 are considered under a plane stress state. The simulations were run in the implicit static scheme with 20 increments. We compared standard FEM and MM simulations in parallel (with one processor per subdomain for all cases), and found that results were overall comparable (see Fig. 10B,C,D,E for the distribution of the von Mises stress at the final time and Fig. 10F for the prescribed displacement-reaction force curves). Fig. 10G shows the ratio between the magnitude of the fictitious-real displacement discrepancy to real displacement magnitude at shared nodes between processors in the compressed region behind the foreleg (see the dash box in 10A). It is found that the displacement discrepancy is very small even without the mesh refinement. The displacement discrepancy at the extremities vanishes to a computational rounding error as expected since the Kronecker delta property is satisfied at the vertices of the MM domains. Once the mesh refinement is applied, both the Refined and Boundary-Refined cases successfully eliminate the boundary discrepancies. This significant finding demonstrates that the computational inaccuracies caused by the proposed approach of truncating nodal neighbourhood at the partitions’ boundaries can be successfully eliminated by shifting the relative contribution of boundary and bulk nodes within the support domain. Note that the results also show that, even without additional refinement, the proposed parallel MM scheme produces remarkably quantitatively accurate results when compared to FEM simulations.

### 3.4 Performance analysis in parallel simulations

The numerical simulations can run in parallel, in which a number of processors compute simultaneously to increase the computational power and/or reduce significantly the computational time. To evaluate the ability of a parallel programme to do so, scaling (or scalability) analyses are widely used. In this section, the scaling of our FEM, MM, and coupled FEM-MM schemes in parallel is investigated. The Oxford Advanced Research Computing (ARC) facility was used for this work.

#### 3.4.1 Strong scaling

Strong scaling refers to the performance of an application when the problem size is kept fixed while the number of cores increases [69]. The parallel performance was examined using a benchmark simulation of a compressive block. Unlike FEM, the domain decomposition into multiple processors currently cannot be automatically performed since the MM subdomains are required to be convex in order to exploit the weak Kronecker delta property.

**Fig. 11:**
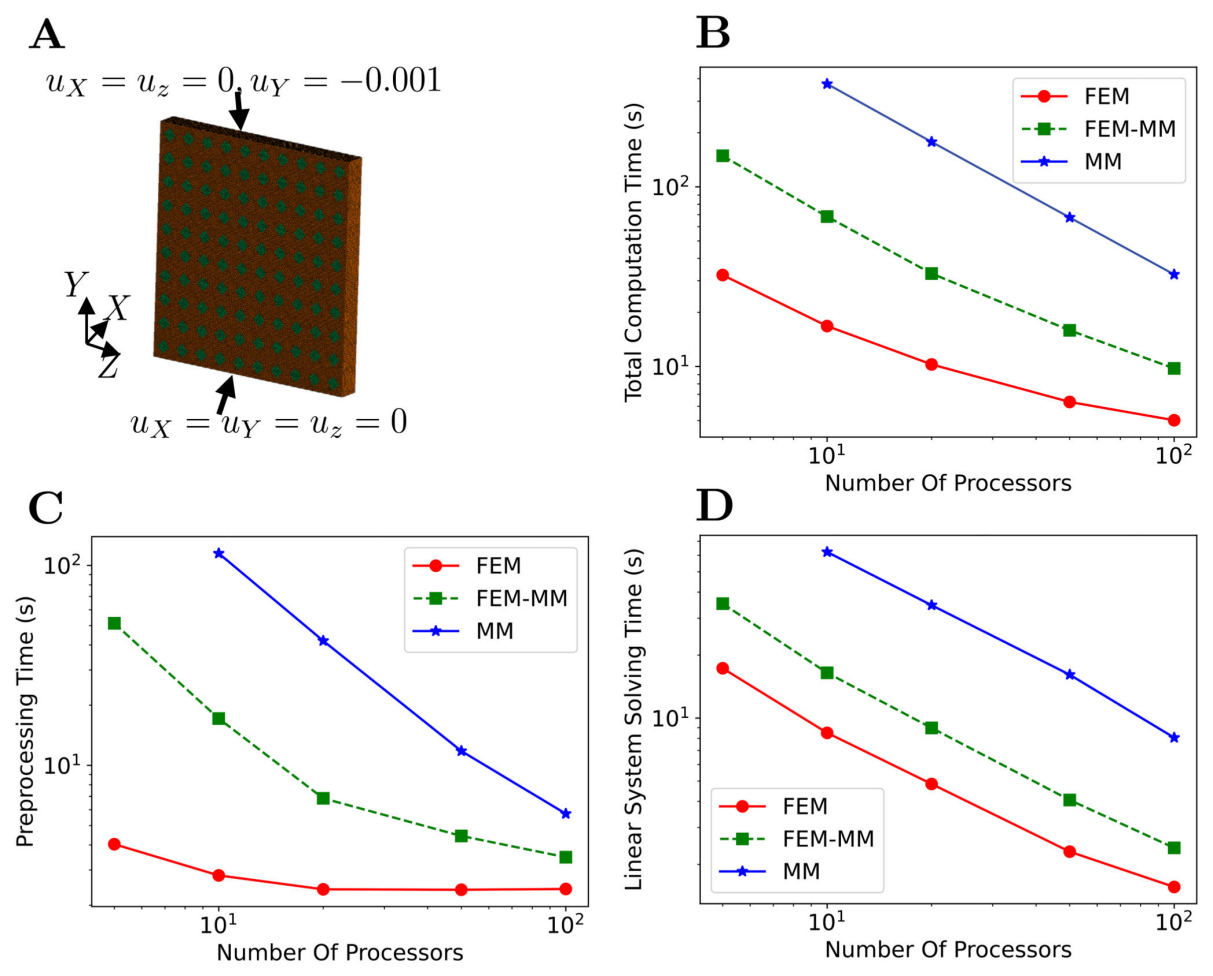
Strong scaling study. (A) Simulation design with the finite element mesh consisting of 549 794 linear tetrahedral elements and boundary conditions. Whenever MM scheme is employed in any subdomain, the mesh corresponds to its MM background one. (B) Scaling performance in terms of total simulation time versus number of cores in a log–log plot. (C) Preprocessing time. (D) Time for solving the linear system with the conjugate gradient iterative solver provided by PETSc [64] in combination with the geometric algebraic multigrid preconditioner. Each simulation is repeated three times and the average value is reported.

The strong scaling performance is investigated using a compression tests of the linear elastic block consisting of 100 unit blocks, see Fig. 11A. The elastic properties *E* = 10^9^ Pa and *v* = 0.3 are used. The linear elastic block is decomposed into 5, 10, 20, 50, or 100 subdomains, which allows performing the parallel simulations in 5, 10, 20, 50, or 100 processors. To facilitate the coupled FEM-MM simulation for comparison purposes, a MM box is considered in the centre of each unit block, see green regions in Fig. 11A. This scenario allows for proper load balancing between processors. In a practical application, a FEM-MM simulation is more advantageous than a MM simulation alone if MM sub-domains are controlled adequately, as the computationally expensive MM partitions decide the computation time of the simulation.

The scaling performance is shown in Fig. 11B in terms of the total computation time versus the number of cores. It is found that the ideal linear scaling can be achieved at low number of processors for all cases. The MM simulations are shown to be computationally expensive because of larger stiffness matrix bandwidth in comparison with the FEM and FEM-MM cases, but the linear scalability can be obtained for a wider range. Fig. 11C shows the preprocessing time. FEM requires much less computational time than MM as expected from the computation of the shape functions, trivial in the case of FEM but expensive for MM. The total time to solve the linear system using PETSc is reported in Fig. 11D where a linear scaling is achieved in the range of number of processors under consideration. Overall, the use of the coupled FEM-MM scheme is less computationally expensive than MM but can leverage the accuracy of the MM scheme in critical regions.

#### 3.4.2 Weak scaling

**Fig. 12:**
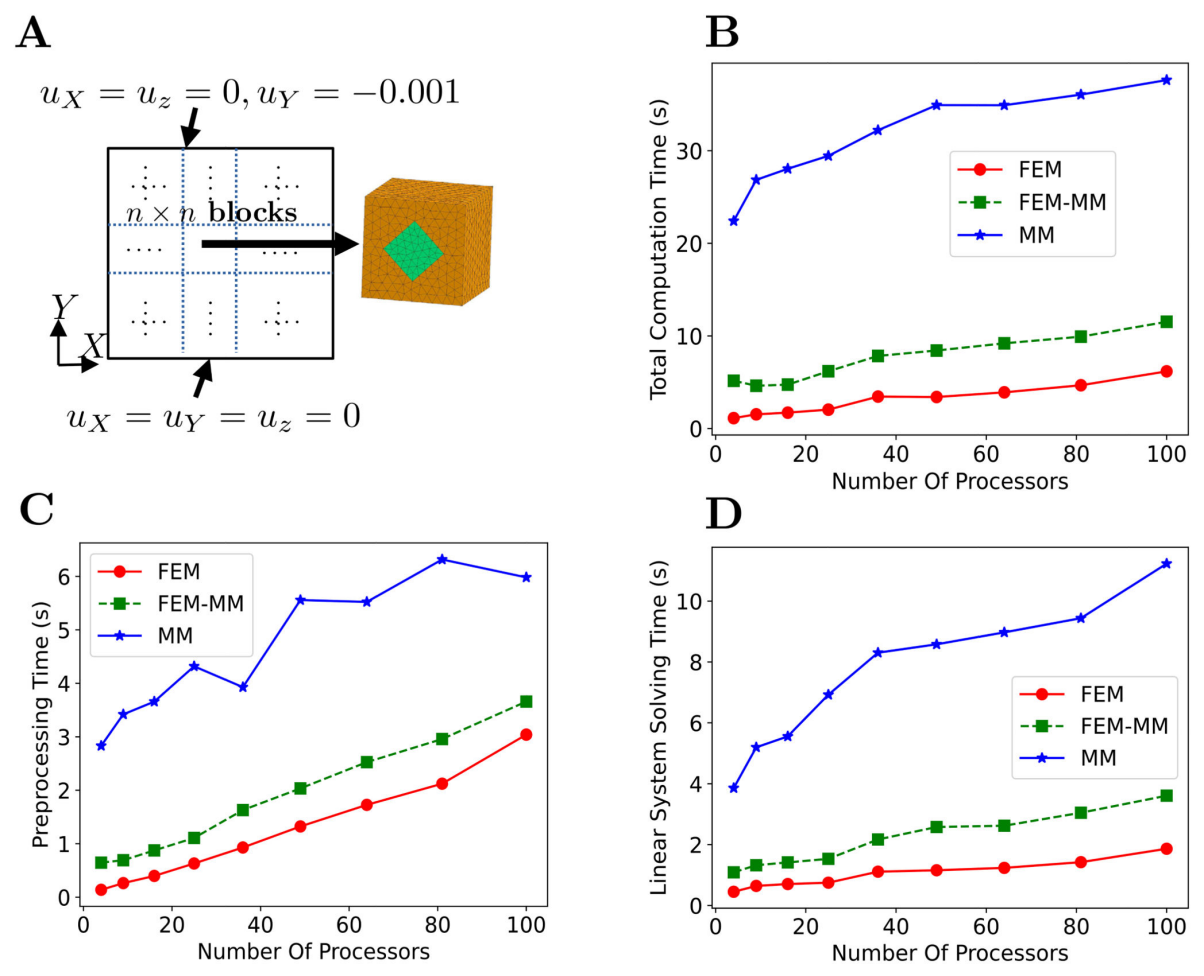
Weak scaling study. (A) Simulation design consisting of *n* × *n* unit blocks where *n* corresponds to the number of cores. Each block containing 5494 linear tetrahedral elements belongs to a processor. (B) Scaling performance in terms of the total simulation time versus number of processors. (C) Preprocessing time. (D) Time for solving the linear system with the conjugate gradient iterative solver provided by PETSc [64] in combination with the geometric algebraic multigrid preconditioner. Each simulation is repeated three times and the average value is reported.

In a weak scaling test, instead of keeping the problem size fixed, we increase the number of cores and the problem size proportionally [70]. The compressive test described in Fig. 11 is considered but with *n* × *n* unit blocks, see Fig. 12A. Each block belongs to one processor, so the problem of *n* × *n* unit blocks can be performed in *n*^2^ processors while the same problem size in each processor is retained. The weak scaling performance in terms of the total computation time versus the number of processors is shown in Fig. 12B when varying *n* from 2 to 10.

In an ideal scenario, the computation time remains constant. However, Fig. 12B shows an increasing computation time with increasing number of processors. This can be explained by (i) the increasing preprocessing time as reported in Fig. 12C arising from loading a larger input file when the problem size increases and (ii) the increase of the computational time required to solve the finite element linear system as reported in Fig. 12D. Clearly, the use of the iterative solver in PETSc in combination with geometric algebraic multigrid preconditioner does not allow for an ideal scaling. However, it can be seen that the coupled FEM-MM scheme is less computationally expensive than the MM alone, thus providing a good balance between MM accuracy and FEM scalability.

### 3.5 Compression test with finite strain elastoplastic behaviour

**Fig. 13:**
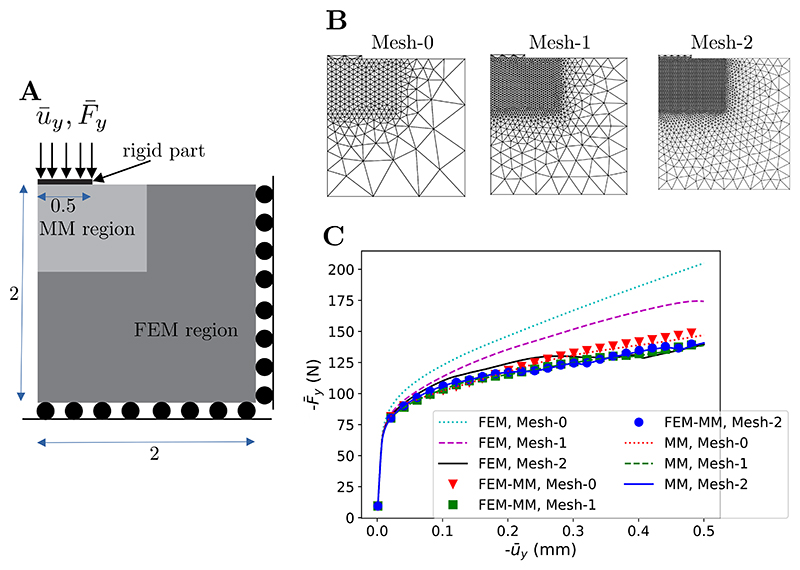
Compression test on an elastoplastic block. (A) Geometry and boundary conditions. (B) Three meshes used in the analysis (576, 2200 and 6195 triangles). (C) Mechanical response in terms of the reaction force versus the prescribed displacement.

In this section, a compression test under plane strain conditions on an elastoplastic block is considered. In this test, a portion of the upper face is pushed downward with a rigid plate, thus creating a localised region of shearing. The geometry and boundary conditions are shown in Fig. 13A. The numerical results for FEM, MM, and coupled FEM-MM schemes are compared. When using the coupled FEM-MM scheme, MM is used for the highly deformed region while FEM is used for the remaining region. The rigid part is modelled by FEM with a linear elastic behaviour and an arbitrary high Young’s modulus. The adaptive strategy to find the value of *α* described in Section 2.2 is used. The finite strain elastoplastic model reported in Appendix is considered using a Young’s modulus of *E* = 20 GPa, Poisson’s ratio of *v* = 0.3, initial yield stress $\sigma_{y}^{0}=100\text{MPa}$, reference equivalent plastic strain *γ*^0^ = 0.005 and *n* = 0.1.

Three different meshes noted Mesh-0, Mesh-1 and Mesh-2 consisting of 576, 2200 and 6195 linear triangles, respectively, are considered, see Fig. 13B. When MM is used, the background meshes used for quadrature points identification are used for visualisation purposes. The mechanical responses in terms of the vertical reaction force ${\bar{F}}_{y}$ versus the prescribed displacement ${\bar{u}}_{y}$ are shown in Fig. 13C. It can be seen that FEM results are mesh dependent results while the MM and coupled FEM-MM schemes are converged already with the coarsest mesh Mesh-0. FEM requires a much finer mesh to achieve similar results to MM and FEM-MM. The use of MM domains in high deformation regions improves significantly the numerical accuracy.

**Fig. 14:**
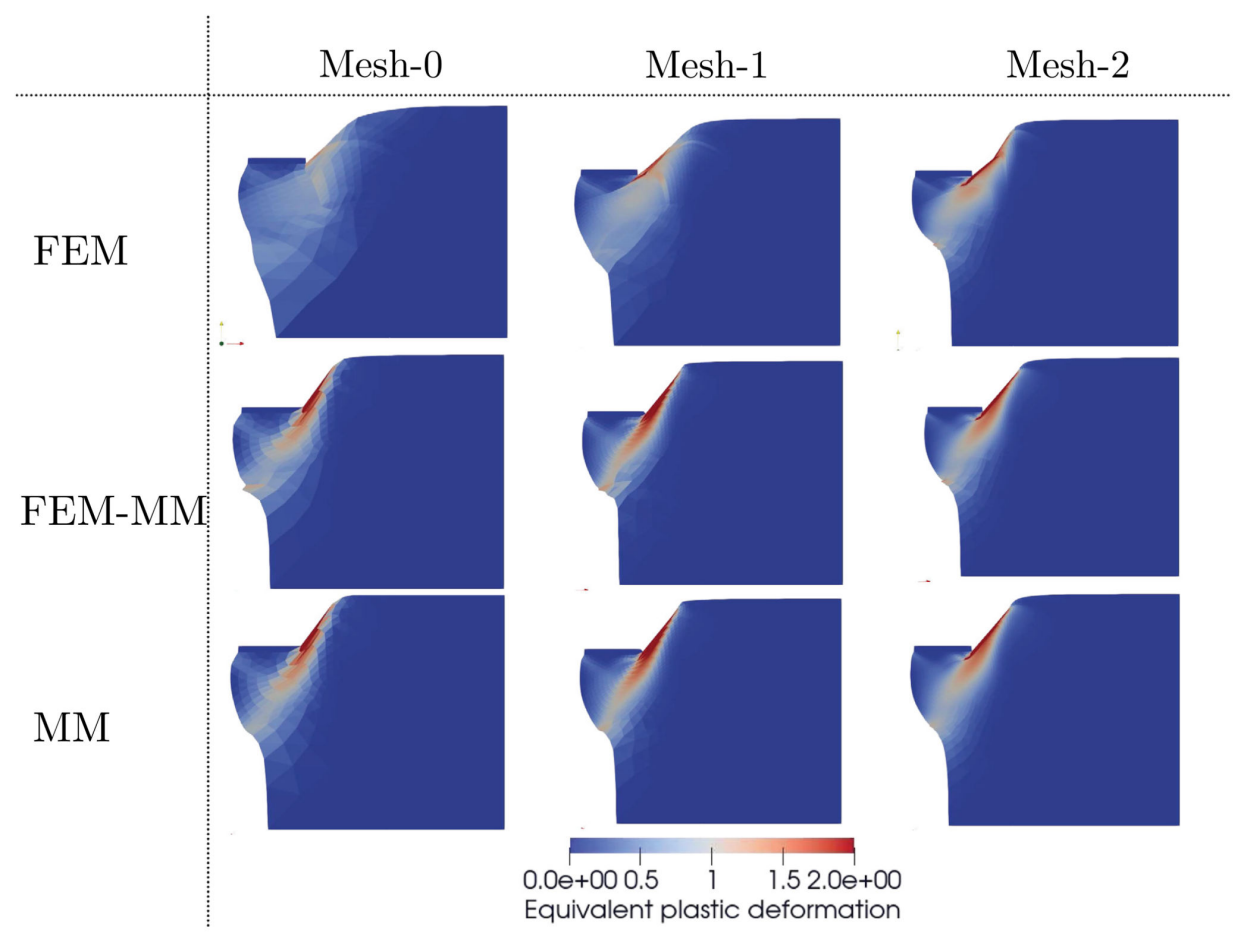
Compression test on an elastoplastic block. Distribution of the equivalent plastic deformation at the end time. Mesh-0, Mesh-1, and Mesh-2 are shown in Fig. 13B.

Fig. 14 shows the distribution of the plastic deformation for the different meshes and simulation schemes. It is found that FEM provides poor solutions with Mesh-0 while the FEM-MM and MM schemes provide better results at equivalent level of discretisation. The computational time is reported in Table 1 for a sequential calculation on an Intel i9 10th Generation core. The numerical simulations only considering MM are computationally expensive while the use of FEM in non-critical regions allows for a reduction of the cost with the benefit of a much better accuracy.

## 4 Conclusion

In this paper, we presented a bespoke numerical programme combining FEM and MM in sequential and parallel simulations. We demonstrated that coupling of FEM and MM regions can significantly improve solutions for structures undergoing large deformations while mitigating computational costs. Furthermore, we demonstrated that MM simulations can be significantly sped up using a simple parallelisation strategy: rather than considering the influence of nodes inbetween neighbouring regions on separate processors, we implemented a framework considering only the influence of shared nodes at the boundary, analogous to parallelisation strategies in FEM. Although we observed minor inaccuracies at shared boundary nodes, this approach produced simulation outcomes that were similar to FEM simulations, suggesting that this strategy can be viable without further modifications in certain simulations. We furthermore demonstrated that boundary inaccuracies could be completely eliminated by selective remeshing avoiding the addition of further nodes on the boundary of two regions assigned to different processors, thus providing a simple strategy to improve computational accuracy when necessary. Our parallelisation approach enabled us to achieve significant speed-ups of both simulation and preprocessing times. Taken together, the approach provides a robust and versatile computational resource to tackle challenging problems in mechanics and biomechanics, especially in problems including growth and rapid large deformations [71]. On a final note, as our MM is considered in a Lagrangian framework, the cloud of influence of any point depends on the current configuration. While it has the advantage to depend solely on the convexity of the reference configuration, the radius of influence of each point needs to be defined in the current configuration, leading to the need to regularly “refresh” the cloud of influence, similarly to FEM remeshing requirements.

## Supplementary Material

Appendix

## Figures and Tables

**Table 1:** Computation time (in seconds) in the compression test with FEM, MM, and coupled FEM-MM schemes.

	Mesh-0	Mesh-1	Mesh-2
FEM	363	1740	4826
FEM-MM	1689	8573	21,398
MM	2349	10,411	25,011

## Data Availability

The *MuPhiSim* (Multiphysics Simulation Framework) suite is available under academic license on https://github.com/muphisim.

## Footnotes

2 For elastic materials, Eq. (5) becomes ***P*** (*t*) = **𝒫** (***F*** (*t*)). For history-dependent materials, Eq. (5) is generally rewritten as ***P*** (*t*) = **𝒫** (***F*** (*t*) ; ***Z*** (*t*)) and evolution laws for ***Z***, where ***Z*** is a vector including all history-dependent variables.
