# Supplementary material for: A numerical framework coupling finite element and meshless methods in sequential and parallel simulations
Source: Finite Elem Anal Des. Author manuscript; Available in PMC 2025 Jul 21. (PMC7617925; doi:10.1016/j.finel.2023.103927)
Supplement: Appendix [file EMS206756-supplement-Appendix.pdf]

Appendix. Finite strain J_2 plasticity model

The finite strain J_2 elastoplastic constitutive model used here was proposed in Ref. [72] and is briefly summarised here. The deformation gradient $\boldsymbol{F}$ can be decomposed into an elastic part $\boldsymbol{F}^e$ and a plastic part $\boldsymbol{F}^p$ as follows

$$\boldsymbol{F} = \boldsymbol{F}^e \cdot \boldsymbol{F}^p \,. \tag{20}$$

Incompressible plastic deformation is assumed, i.e., $\det \boldsymbol{F}^p = 1$. The following bi-logarithmic elastic potential is considered here:

$$\Psi\left(\boldsymbol{C}^e\right) = \frac{K}{2} \ln^2 J + \frac{\mu}{4} \operatorname{dev}\left(\ln \boldsymbol{C}^e\right) : \operatorname{dev}\left(\ln \boldsymbol{C}^e\right) \,, \tag{21}$$

where $J = \det \boldsymbol{F}$, $\boldsymbol{C}^e = \boldsymbol{F}^{eT} \cdot \boldsymbol{F}^e$, $K = \frac{E}{3(1-2\nu)}$ and $\mu = \frac{E}{2(1+\nu)}$ with E and ν being, respectively, the Young's modulus and Poisson's ratio, and $\operatorname{dev}(\bullet)$ is the deviatoric operator. The first Piola–Kirchhoff stress can be derived as

$$\boldsymbol{P} = K \boldsymbol{F}^{-T} \ln J + \mu \boldsymbol{F}^e \cdot \left[\boldsymbol{C}^{e-1} \cdot \operatorname{dev}\left(\ln \boldsymbol{C}^e\right)\right] \cdot \boldsymbol{F}^{p-T} \,. \tag{22}$$

The Kirchhoff stress is computed by $\boldsymbol{\tau} = \boldsymbol{P} \cdot \boldsymbol{F}^T$, leading to

$$\boldsymbol{\tau} = K \ln J \boldsymbol{I} + \mu \boldsymbol{F}^e \cdot \left[\boldsymbol{C}^{e-1} \cdot \operatorname{dev}\left(\ln \boldsymbol{C}^e\right)\right] \cdot \boldsymbol{F}^{eT} \,. \tag{23}$$

The plasticity criterion follows the J_2 plasticity theory as

$$f = \tau_{eq} - \sigma_y^0 - R(\gamma) \leq 0 \,, \tag{24}$$

where $\tau_{eq} = \sqrt{\frac{3}{2} \operatorname{dev}(\boldsymbol{\tau}) : \operatorname{dev}(\boldsymbol{\tau})}$ is the von Mises equivalent stress, σ_y^0 is the initial yield stress, and $R \geq 0$ is the isotropic hardening stress,

function of the equivalent plastic strain γ. In this work, an isotropic hardening law is considered:

$$R = \sigma_y^0 \left(1 + \frac{\gamma}{\gamma^0}\right)^n - \sigma_y^0 \,. \tag{25}$$

where γ^0 is the reference equivalent plastic strain and n is a material parameter. The evolution law for $\boldsymbol{F}^p$ follows the normality rule

$$\dot{\boldsymbol{F}}^p = \dot{\gamma} \boldsymbol{N} \cdot \boldsymbol{F}^p \,, \tag{26}$$

where $\boldsymbol{N}$ is the normal to the yield surface. The resolution of the system of Eqs. (20)–(26) follows a predictor–corrector scheme as detailed in Ref. [72].
